# Differential Change in Hippocampal Radial Astrocytes and Neurogenesis in Shorebirds With Contrasting Migratory Routes

**DOI:** 10.3389/fnana.2019.00082

**Published:** 2019-09-25

**Authors:** Camila Mendes de Lima, Patrick Douglas Corrêa Pereira, Ediely Pereira Henrique, Marcus Augusto de Oliveira, Dario Carvalho Paulo, Lucas Silva de Siqueira, Daniel Guerreiro Diniz, Diego Almeida Miranda, Mauro André Damasceno de Melo, Nara Gyzely de Morais Magalhães, David Francis Sherry, Cristovam Wanderley Picanço Diniz, Cristovam Guerreiro Diniz

**Affiliations:** ^1^Laboratório de Investigações em Neurodegeneração e Infecção no Hospital Universitário João de Barros Barreto, Instituto de Ciências Biológicas, Universidade Federal do Pará, Belém, Brazil; ^2^Laboratório de Biologia Molecular e Neuroecologia, Instituto Federal de Educação Ciência e Tecnologia do Pará, Campus Bragança, Bragança, Brazil; ^3^Advanced Facility for Avian Research, Department of Psychology, University of Western Ontario, London, ON, Canada

**Keywords:** radial astrocytes, morphometry, hippocampus, neurogenesis, shorebirds, *Calidris pusilla*, *Charadriussemipalmatus*

## Abstract

Little is known about environmental influences on radial glia-like (RGL) α cells (radial astrocytes) and their relation to neurogenesis. Because radial glia is involved in adult neurogenesis and astrogenesis, we investigated this association in two migratory shorebird species that complete their autumnal migration using contrasting strategies. Before their flights to South America, the birds stop over at the Bay of Fundy in Canada. From there, the semipalmated sandpiper (*Calidris pusilla*) crosses the Atlantic Ocean in a non-stop 5-day flight, whereas the semipalmated plover (*Charadrius semipalmatus*) flies primarily overland with stopovers for rest and feeding. From the hierarchical cluster analysis of multimodal morphometric features, followed by the discriminant analysis, the radial astrocytes were classified into two main morphotypes, Type I and Type II. After migration, we detected differential changes in the morphology of these cells that were more intense in Type I than in Type II in both species. We also compared the number of doublecortin (DCX)-immunolabeled neurons with morphometric features of radial glial–like α cells in the hippocampal V region between *C. pusilla* and *C. semipalmatus* before and after autumn migration. Compared to migrating birds, the convex hull surface area of radial astrocytes increased significantly in wintering individuals in both *C. semipalmatus* and *C. pusilla*. Although to a different extent we found a strong correlation between the increase in the convex hull surface area and the increase in the total number of DCX immunostained neurons in both species. Despite phylogenetic differences, it is of interest to note that the increased morphological complexity of radial astrocytes in *C. semipalmatus* coincides with the fact that during the migratory process over the continent, the visuospatial environment changes more intensely than that associated with migration over Atlantic. The migratory flight of the semipalmated plover, with stopovers for feeding and rest, vs. the non-stop flight of the semipalmated sandpiper may differentially affect radial astrocyte morphology and neurogenesis.

## Introduction

Radial glia are non-neuronal cells of the astroglial lineage, characterized by an ovoid cell body located near the ventricular wall with typically asymmetric bipolar branches. The shorter ends of the branches extend toward the ventricular wall and the longer ends in the opposite direction, toward the pia (Parnavelas and Nadarajah, 2001). Rakic coined the name when describing the mode of neuronal migration to the superficial layers of the fetal monkey neocortex (Rakic, 1972), suggesting that glial radial fibers provided guides for cell migration. Further neuronal migration during central nervous system development and adult neurogenesis has been described more recently. Indeed, immature neurons depart toward their final destination from their neurogenic niches guided not only radial astrocytes (Marin and Rubenstein, 2003; Rakic, 2003) but also by neuronal axons (Hutchins et al., 2013) and blood vessels as scaffolds (Segarra et al., 2015).

The true (primary) radial glia seem to be limited to the developing brain, which has led to a change in the original designation of adult radial astrocytes to “radial glia-like (RGL)” in the adult brain. Thus, adult radial glia, RGL cells, or radial astrocytes were coined to limit radial glia designation to the developmental period (Verkhratsky and Nedergaard, 2018). RGL cells expressing glial acid fibrillary protein (GFAP) found in the subgranular layer of the dentate gyrus and subventricular zone of adult animals show increased proliferative activity in association with vascular interaction, voluntary exercise on running wheels, or enriched environment (Bednarczyk et al., 2011; Kempermann, 2012).

Results obtained with confocal microscopy revealed that radial astrocytes can be classified from their morphology into two main types (Gebara et al., 2016). The most common type designated α cell shows long and sparse processes when compared to the less frequent and less branched type designated β cell. Only alpha cells have proliferative capacity and give rise to neurons and astrocytes (Gebara et al., 2016). In adult animals of all vertebrates, neurogenesis persists in limited areas and radial astrocytes are central elements of these neurogenic niches (Falk and Götz, 2017; Augusto-Oliveira et al., 2019; Oppenheim, 2019).

In adult birds and mammals (Alvarez-Buylla et al., 1987) neurogenic niches co-exist with radial astrocytes and are found mainly in the forebrain (Dimou and Götz, 2014). Indeed, in adult animals, the subependymal layer aligned with the ventricular ependyma contains the cell bodies of actively dividing radial astrocytes (Dimou and Götz, 2014). Observations in adult birds including zebra finch, quail, chicken and canaries have shown that radial astrocytes have their cell bodies located in the ventricular zone of the lateral forebrain ventricles with radial fibers penetrating the forebrain parenchyma of up several millimeters (Alvarez-Buylla et al., 1987).

Previous studies involving comparative neuroanatomy (Krebs et al., 1989; Sherry and Vaccarino, 1989; Jacobs et al., 1990; Healy and Krebs, 1996; Garamszegi and Eens, 2004; Lucas et al., 2004; Roth and Pravosudov, 2009; LaDage et al., 2010, 2011; Garamszegi and Eens, 2004), hippocampal lesions (Sherry and Vaccarino, 1989; Hampton and Shettleworth, 1996; Colombo et al., 2001) and single-cell recording (Siegel et al., 2005; Hough and Bingman, 2008) has shown that the hippocampus contributes significantly to learning and spatial memory. The contribution of the hippocampus to learning and memory, in turn, seems to be intrinsically associated with the fact that the hippocampus is a neurogenic niche. In fact, the hippocampus remains a neurogenic niche in adulthood (LaDage et al., 2011) and its integrity seems to be essential for long term memory formation, both in birds and mammals (Sherry and Vaccarino, 1989; Oppenheim, 2019). Because migratory behavior has been specifically associated with adult neurogenesis (LaDage et al., 2011; Barkan et al., 2016) and this is consistent with the fact that migratory birds show better long-term memory (Mettke-Hofmann and Gwinner, 2003) and better spatial memory (Cristol et al., 2003; Pravosudov et al., 2006) than non-migrating birds, it may be important to search for potential correlations between radial astrocytes, hippocampal neurogenesis and migratory behavior.

Hippocampal adult neurogenesis may be influenced by several factors including cognitive activity, environmental enrichment, exercise, diet, gonadal hormones, and aging (Barnea and Pravosudov, 2011; Galea et al., 2013; Aimone et al., 2014; Oomen et al., 2014; Cameron and Glover, 2015; LaDage, 2015). Losing and acquiring memories are associated with enhanced hippocampal neurogenesis and this seems to be essential for spatial learning and memory associated with migration in birds (LaDage et al., 2011). The new neurons encode new learning and memories (Nottebohm, 2002) contributing to the remodeling of hippocampal circuits (Doetsch and Hen, 2005) which consolidate or delete memories (Frankland et al., 2013). In a previous report, we demonstrated that in adult migratory semipalmated sandpipers (*Calidris pusilla*), autumn migration towards the South American coastline affects neurogenesis (Magalhaes et al., 2017).

Studies in mutant mice with radial glial defects have revealed corresponding defects in neuronal migration suggesting that these cells appear to provide an adequate framework to guide neurons toward their targets (Götz et al., 1998; Pinto and Götz, 2007). In addition, RGL α cells are putative stem cells involved in astrogenesis and neurogenesis, and radial neuronal migration along radial glia fibers has been detected in both immature and adult brain (Scott et al., 2012; Lever et al., 2014; Sun et al., 2015; Bonaguidi et al., 2016; Gebara et al., 2016; Falk and Götz, 2017; Berg et al., 2018). Furthermore, RGL α cells share common molecular signatures with GFAP-immunolabeled astrocytes (Scott et al., 2012; Renzel et al., 2013; Matsue et al., 2018).

To study potential connections between radial astrocytes morphology and neurogenesis we selected two species of shorebirds with very different autumnal migratory routes: one transatlantic long-term uninterrupted flight (*Calidris pusilla*) and another one mostly overland, with many stopovers for feeding and resting (*Charadrius semipalmatus*).

These birds migrate northwards to the arctic and subarctic regions of North America during the breeding season. These shorebirds reproduce in areas between Alaska and Nova Scotia (Sick, 1997). Due to great availability of food, Northern Brazil and the northeast of Pará State are important wintering sites for many of these birds. They accumulate energy to be spent during the migration back to the Arctic in these sites and find suitable climate for moulting. A variety of species of migratory shorebirds including the semipalmated plover *C. semipalmatus* and the semipalmated sandpiper *C. pusilla* chose the coast of Bragança for wintering.

A variety of navigational systems are combined with visuospatial learning and memory in migratory birds to maintain orientation during flight (Wiltschko et al., 2013; Mouritsen et al., 2016). For example, birds can use the Earth’s geomagnetic field as a compass to keep their course during their migratory flight using cryptochrome magnetoreception (Lau et al., 2012; Fusani et al., 2014) and the hippocampus has been proposed as an integrative center for all environmental navigational information (Mouritsen et al., 2016).

We assumed that the visuospatial recognition tasks of the contrasting pathways of *C. pusilla* and *C. semipalmatus* migratory flights would impose differential changes in radial astrocyte morphology and neurogenesis. We searched for potential correlations between the number of doublecortin immunolabeled neurons of the hippocampal formation (neurogenesis) and morphological features of 3D microscopically reconstructed radial astrocytes from the V-shaped hippocampal area. This region shows mouse-like conserved gene expression patterns, confirming a homology previously suggested with the mammalian dentate gyrus (Gupta et al., 2012; Atoji et al., 2016).

## Materials and Methods

The captures of the individuals used in this study were authorized through licenses No 44551-2 from the Chico Mendes Institute for Conservation and Biodiversity (ICMBio) and permission for Scientific Capture ST2783 from the Canadian Wildlife Service. Experimental manipulation followed the recommendations expressed by the United States National Health Institute and the Brazilian National Council for Animal Experimentation (CONCEA) with the approval of the subcommittee of the University of Western Ontario and of the Federal University of Pará. Systematic efforts were done to minimize animal discomfort.

We compared the radial astrocyte morphology of five individuals of *C. pusilla* and five of *C. semipalmatus* captured in the Bay of Fundy, Canada (45° 50′19.3” N and 64° 31′5.39” W) in the autumn migration period with five individuals of each species caught in the wintering period on Isla Canela, on the coast of Bragança, Brazil (00°47′09.07 “S and 46°43′11.29” W; 20 individuals in total). All migrating birds (Bay of Fundy, Canada) were captured in August, whereas collecting times for the wintering birds (Isle Canela, Brazil) spanned between August and May (for details, see “Supplementary Material”). Thus, we should keep in mind that potential environmental changes over wintering period may impose methodological limitations to this study.

### Histological and Immunohistochemical Procedures

After capture, the subjects were euthanized with anesthetic overdose and perfused with aldehyde fixatives. After fixation, the brains were cut into a vibratome and 80 μm thick sections were systematically collected to obtain 6 anatomical series with intervals 1:6 between sections. Following antigenic recovery with 0.2 M boric acid pH (9.0), at 70°C, 1 h, and blocking nonspecific antigenic sites, the sections were immunostained for GFAP or doublecortin (DCX), to identify radial astrocytes and new neurons respectively. For this we used anti-GFAP antibody SC-6170 and anti-Doublecortin C-18, SC-8066; Santa Cruz Biotechnology, respectively, diluted 1:500 in 0.05M phosphate buffer saline triton—PBST 0.3%. The process of antigenic recovery and immunolabeling with primary antibodies was done under continuous and gentle agitation for 3 days at 4°C. The sections were then exposed overnight to secondary antibody (Biotinylated Horse Anti-Goat IgG Antibody, BA-9500, Vector Laboratories) diluted 1:400 in PBST. To eliminate endogenous peroxidase, the sections were then incubated in 0.3% hydrogen peroxide in PBS for 15 min. After washing in 0.1 M phosphate buffer pH 7.2–7.4, sections were incubated in avidin-biotin-peroxidase complex (ABC, Vector Laboratories, Burlingame, CA, USA; 37.5 μL of A, 37.5 μL of B in 13.12 ml of 0.3% PBST) for 60 min. After washing in PBS, sections were reacted using the glucose-oxidase-DAB-nickel method (Shu et al., 1988) to view DCX or GFAP-immunolabeled glia. Reaction was interrupted after visualization of the thinner branches of the radial astrocytes and the primary dendrites of the DCX-labeled cells. The sections were then mounted on gelatinized slides and allowed to dry at room temperature. Dehydration in a series of increasing concentrations of alcohols and diaphanization by immersion in two xylene baths were done. Finally, the sections were coverslipped with Entellan (Sigma-Aldrich). To confirm the specificity of the reaction, some sections went through the same immunolabeling steps without the addition of the primary antibody (Saper and Sawchenko, 2003). As expected, no radial astrocyte or new neuron were detected in the control sections. Further details of immunostaining processing can be found in previous publications (Carvalho-Paulo et al., 2017; Magalhaes et al., 2017).

### Stereology: DCX-Positive Neuronal Numbers

Histological changes, shrinkage, or damage-induced expansion of tissue may change significantly counting results (West et al., 1991). To avoid potential influence of these factors on our findings we adopted the optical fractionator methodology that seems to be unaffected by those variables (West, 1999).

In reacted sections for doublecortin immunolabeling, the limits of the hippocampal formation of *Calidris pusilla* and *Charadrius semipalmatus* species were digitized at the microscope with 4.0× objective. In the six sections of each animal of each experimental group, the contours of the area of interest for different rostro-caudal levels of hippocampal formation were outlined. Motorized stage (MAC6000, Ludl Electronic Products, Hawthorne, NY, USA) and an optical microscope (Eclipse 80i, NIKON, Japan) with analog-to-digital converters were used to record the spatial coordinate information (X, Y, Z) of each digitized point. This system was coupled to a computer that controlled stage movements with the aid of specialized software (Stereoinvestigator, MicroBrightField, Williston, VT, USA) to store and analyze the coordinates of the digitized points of interest. In order to avoid ambiguity in identifying objects of interest and ensuring they were within the allowed counting planes, the 4.0× objective was replaced by a 100× plan fluoride objective (NIKON, NA = 1.3, DF = 0.19) used for all counts undertaken.

To estimate the number of DCX immunostained neurons a systematic and random distribution of counting blocks within a series of rostro-caudal sections containing the region of interest was done. The experimenter defined the boundaries of the region of interest and informed to the program the three dimensions of the counting box (width, length and height), the spacing between them (grid), and the guard zones (distance between the surface of the section to the top of the counting boxes). After filling in this information, the program displays the spatial arrangement of the boxes relative to the section contour from the filled parameters and automatically changes the slide position to start a new count in a new box every time the experimenter closes the previous box count.

In each counting box, the relative thickness of the section defined by the interval between its lower and upper surfaces was carefully defined. For this we used the microscope’s fine focus, defining the point immediately out of focus above (top of the section) and below (bottom). At the end of counting all the boxes in each section, the program estimates the total number of cells in that section, generates the total number of cells marked by the experimenter and the expected total estimate of cells for the section with its respective coefficient of error (CE). The latter is used as an indicator to assess whether the dimensions and number of boxes are adequate to obtain average values representative of the total number of cells in the structure under analysis. CE greater than 0.05 indicate in most cases, methodological error and the need for changes in the chosen parameters. Because the thickness and distribution of cells in the section were uneven, we estimated the total number of cells based on the weighted section thickness (Gundersen and Jensen, 1987).

The experimental parameters and counting results in the region of interest are shown for each bird in the supplementary materials. The grid size used was adapted to achieve an acceptable CE. The calculation of the CE for the total DCX-neuron count in each bird used in the present study adopted the one-stage systematic sampling procedure (Schaeffer CE) that has been previously validated (Gundersen and Jensen, 1987; Glaser and Wilson, 1998). The grid size used was adapted to achieve an acceptable CE. The calculation of the CE for the total DCX-neuron count in each bird used in the present study adopted the one-stage systematic sampling procedure (Schaeffer CE) that has been previously validated (Glaser and Wilson, 1998).

The estimation of the total number of cells was obtained in the present work by using the optical fractionator which is based on three sample fractions: the thickness sampling fraction; the area sampling fraction and the section sampling fraction. Thus, the total number of cells is defined by the following equation:

N=EQ*1/ssf*1/asf*1/tsf,

where *N* is the total number of cells, EQ is the number of counted cells, *ssf* is the ratio between the number of sections counted and the total number of sections containing the area of interest, *asf* is the ratio between the counting box area and the grid area, and *tsf* is the ratio between the counting box height and the section thickness. The product of these three fractions corresponds to the total estimated cells (West et al., 1991).

Further experimental parameters and counting results are exhibited on Supplementary Tables S1–S6.

### Three-Dimensional Radial Astrocyte Reconstruction

**Figure 1:**
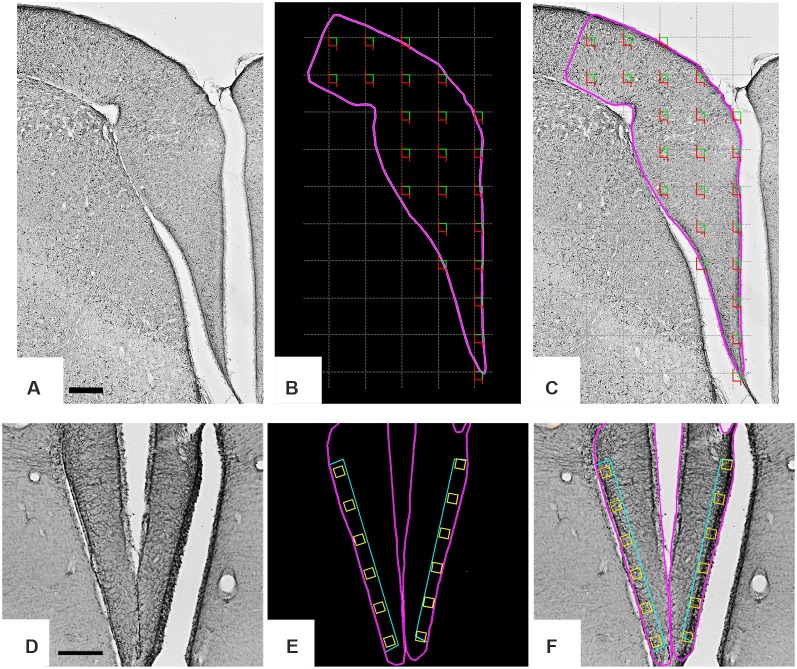
Low-power photomicrographs of the *C. semipalmatus* hippocampal formation from a section immunolabeled with anti-glial acid fibrillary protein (GFAP) antibody to define the limits of the area of interest and the sampling strategy. A random and systematic sampling approach was applied to the hippocampal formation to count cells **(A–C)** and to the margin of the hippocampal V area to select radial astrocytes for 3D reconstruction **(D–F)**. The hippocampal formation is shown inside the pink contour (B). The gray grid **(C)** establishes the intervals between the square green/red counting boxes and illustrates the systematic random sampling approach. Green and red lines of the counting boxes define permitted and prohibited counting lines. A single radial astrocyte located inside every yellow box **(E,F)** aligned with the hippocampal V area was selected for 3D reconstruction. Scale bars: **(A–C)** = 250 μm; **(D–F)** = 500 μm.

Neurolucida Neuron Tracing Software (Neurolucida 11.03; MBF Bioscience, Williston, VT, USA) was used to acquire and analyze images. Based on previous evidence of 75% shrinkage (Carlo and Stevens, 2011), the Z-axis of our 3D reconstructions was linearly corrected. Only cells with unequivocally complete arbors were included in the 3D analysis. We performed digital 3D reconstruction of 1,068 radial astrocytes (494 from *C. pusilla* and 574 from *C. semipalmatus*) selected from five migrating and five wintering birds for each species (Figure 1).

The boundaries of the hippocampal formation comprised hippocampus proper and the parahippocampal area (Atoji and Wild, 2006; Diniz et al., 2016). Only radial astrocytes from the hippocampal V area (Figures 1D–F) were used for 3D reconstruction. As previously mentioned this region shows mouse-like conserved gene expression patterns, confirming a homology previously suggested with the mammalian dentate gyrus (Gupta et al., 2012; Atoji et al., 2016).

The statistical analysis of radial astrocyte morphology was based on 15 morphometric variables extracted from 3D reconstructions of radial astrocytes of the hippocampal V region of four experimental groups: two species (*C. semipalmatus* and *C. pusilla*) and two capture sites (Brazil and Canada). The capture in the Bay of Fundy (Canada) corresponded to that of the migrating birds and the capture made on Isla Canela (Brazil) corresponded to the birds in the wintering period. From each experimental group, we reconstructed the following numbers of radial astrocytes: *C. pusilla*, Canada, 252 and Brazil, 242; *C. semipalmatus*, Canada, 235 and Brazil, 339.

This analysis preliminarily required the detection of which of the 15 morphometric variables in each experimental group showed at least a bi-modal distribution. For this purpose, we estimated the multimodality index for each one. As previously recommended (Schweitzer and Renehan, 1997), for cluster analysis, we used all morphometric quantitative variables with multimodality indices (MMIs) greater than 0.55 that included all animals from each group. We estimated the MMI based on the skewness and kurtosis of our sample for each morphometric variable as:

$$MMI=[M3^{2}+1]/[M4+3{\left(n-1\right)}^{2}/(n-2)(n-3)]$$

where M3 is skewness, M4 is kurtosis, and *n* is the sample size (Kolb et al., 1994; Schweitzer and Renehan, 1997). The following multimodal variables were used for cluster analysis:

- *C. pusilla* (Canada): total branch length; segments/mm; number of trees; complexity; and convex hull volume, surface, area, and perimeter
- *C. pusilla* (Brazil): total branch length; average branch length; tortuosity; branch volume; number of segments; segments/mm; surface area; complexity; and convex hull, surface, area, and perimeter
- *C. semipalmatus* (Canada): average branch length; complexity; and convex hull volume, surface, and area
- *C. semipalmatus* (Brazil): average branch length; branch volume, surface area; complexity; and convex hull volume, surface; area, and perimeter

Each of the four datasets of multimodal morphometric features was submitted to hierarchical cluster analysis. The results indicated the classification of radial astrocytes into two large groups (Type I and Type II) in each of the four data sets. The identification of cell types using this methodology has helped in understanding cell structure and function in nuclei and areas of the central nervous system where cell morphologies are not readily distinguishable (Schweitzer and Renehan, 1997). We used Ward’s method with standardized variables and a dendrogram to illustrate the morphological classification.

We used discriminant analysis to identify the morphometric variables that most contributed to distinguish radial astrocytes morphotypes suggested by the cluster analysis. Parametric *t*-tests and non-parametric Mann–Whitney *U* tests were used to detect differences between datasets showing equal and unequal variances, respectively. Analysis of variance (ANOVA)/Kruskal–Wallis testing for independent groups was also used to compare clusters within each group and between groups and to detect possible morphological differences between average values for morphometric features of our radial astrocyte samples from the *C. pusilla* and *C. semipalmatus* hippocampal V area.

For the choice of the representative cell of each group, which illustrates the cluster cell types, a distance matrix was used to obtain the sum of the distances of each cell relative to all others. We postulated that the cell that best represents each group would have the smallest sum of distances. The matrices were constructed with the combination of all cells of a given group taken pairwise, followed by the weighted calculation of a scalar Euclidean distance between cells using all morphometric variables.

## Results

### Area, Object of Interest, and Morphometric Analysis

**Figure 2:**
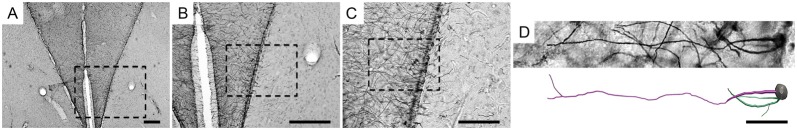
Low- and medium-power photomicrographs of the hippocampal V area **(A–C)** and a high-power 3D photomicrograph and digital reconstruction **(D)** of a radial astrocyte of a *C. semipalmatus* individual, captured on the coast of Bragança, Pará, Brazil. The square dotted line indicates the hippocampal V area containing the reconstructed radial astrocyte. Scale bars: **(A)** = 250 μm; **(B)** = 250 μm; **(C)** = 125 μm; **(D)** = 25 μm.

The GFAP-immunolabeled radial astrocyte in Figure 2 is a cell type of the astroglial lineage, characterized by an ovoid cell body located near the ventricular wall, showing long radial processes in the opposite direction. Figure 2 shows, under different magnifications, details of a GFAP-immunolabeled radial astrocyte placed at the margin of the V hippocampal area, near the ventricle. Under the 100× oil immersion objective, all morphological details and localization of a radial astrocyte for 3D were digitized and stored as x, y, and z coordinates (Figure 2).

Our 3D-reconstructed samples included only radial astrocytes. Individual radial astrocytes were selected using a systematic random sampling approach, and the number of elements selected for reconstruction was rather large, which suggests no *a priori* sampling bias.

**Figure 3:**
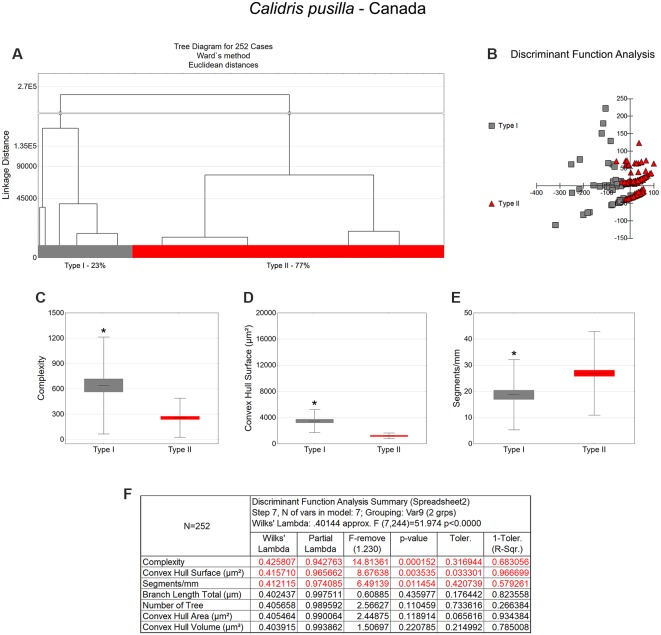
Morphological phenotype groups of astrocytes in the hippocampal V area of *C. pusilla* migrating birds. Cluster discriminant analysis (Ward’s method) was performed after 3D reconstruction of astrocytes from five birds. **(A)** Dendrogram groupings of 252 radial astrocytes identified two main morphological phenotypes, Type I and Type II. **(B,F)** Graphic representations of discriminant function analysis and correspondent summary. Graphic representations of morphological complexity **(C)**, convex hull surface **(D)**, and segments/mm **(E)**, mean values and corresponding standard deviations (whiskers) and errors (gray and red areas); (*) indicates significant differences between Type I and Type II radial astrocytes morphometric features. The variable that contributed the most to cluster formation was complexity (*p* < 0.00015; see discriminant function analysis summary, **F**). Type I astrocytes (gray dots) showed higher x–y dispersion than Type II astrocytes (red dots). Radial astrocytes were reconstructed from both the rostral and caudal regions of the hippocampal formation; cluster analysis was based on multimodal morphometric features of the astrocytes (MMI > 0.55).

**Figure 4:**
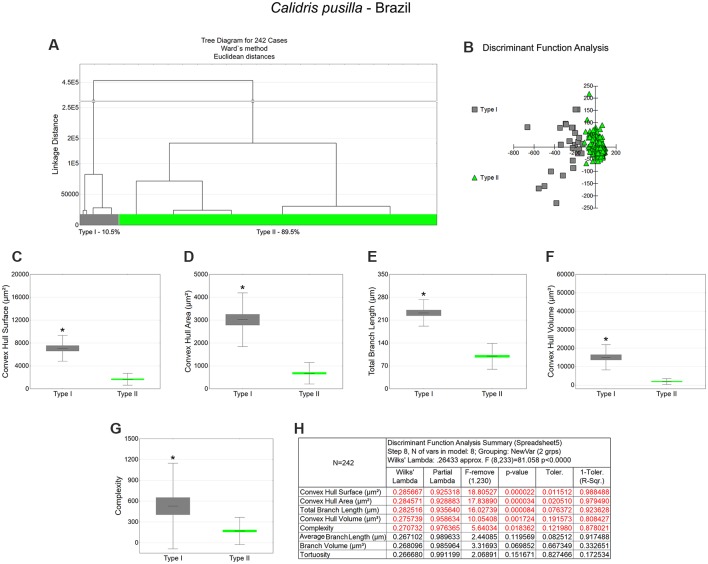
The morphological phenotypes of astrocytes in the hippocampal V area of *C. pusilla* wintering birds. Cluster discriminant analysis (Ward’s method) was performed after 3D reconstructions of astrocytes from five birds. **(A)** Dendrogram groupings of 242 radial astrocytes identified two main morphological phenotypes, Type I and Type II. **(B)** Graphic representation of the discriminant analysis. The variable that contributed the most to cluster formation was convex hull surface (*p* < 0.00002; see discriminant function analysis summary, **H**). Type I astrocytes (gray dots in **B**) showed higher x–y dispersion than type II astrocytes (green dots). Graphic representations of mean values and corresponding standard deviations (whiskers) and errors (gray and green areas) of convex hull surface **(C)**, convex hull area **(D)**, total branch length **(E)**, convex hull volume **(F)**, and complexity **(G)**; (*) indicates significant differences between Type I and Type II radial astrocyte morphometric features. Radial astrocytes were reconstructed from both the rostral and caudal regions of the hippocampal formation; cluster analysis was based on multimodal morphometric features of astrocytes (MMI > 0.55).

**Figure 5:**
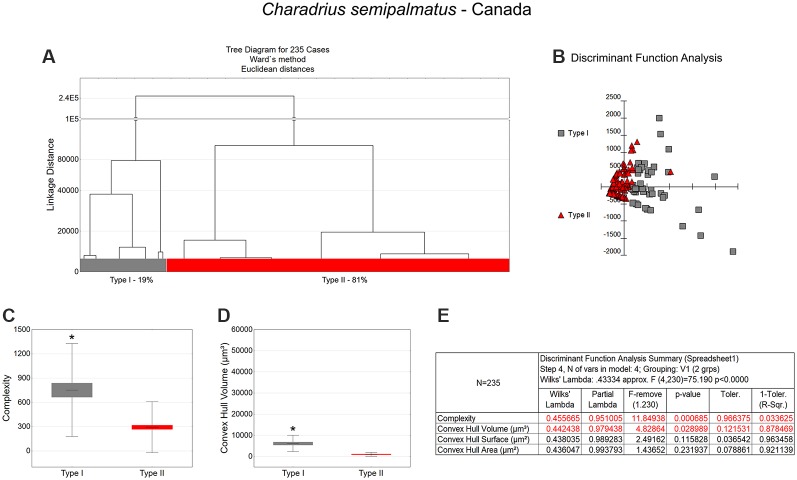
Cluster analysis to classify the morphological phenotypes of radial astrocytes in the hippocampal V area of migrating *C. semipalmatus*. Hierarchical cluster and discriminant analysis (Ward’s method) were performed after 3D reconstructions of radial astrocytes from 5 birds. **(A)** Dendrogram groupings of 235 radial astrocytes identified two main morphological phenotypes, Type I and Type II. **(B)** Graphic representation of discriminant analysis. The variable that contributed the most to cluster formation was complexity (*p* < 0.00068; see discriminant function analysis summary, **E**). Type I astrocytes (gray-filled squares) showed higher x–y dispersion than Type II astrocytes (red-filled triangles). Graphic representations of mean values and corresponding standard deviations (whiskers) and errors (gray and red areas) of morphological complexity **(C)** and convex hull volume **(D)**; (*) indicates significant difference between Type I and Type II radial astrocytes. Radial astrocytes were reconstructed from the rostral to the caudal regions of the hippocampal formation; cluster analysis was based on multimodal morphometric features of the astrocytes (MMI > 0.55).

**Figure 6:**
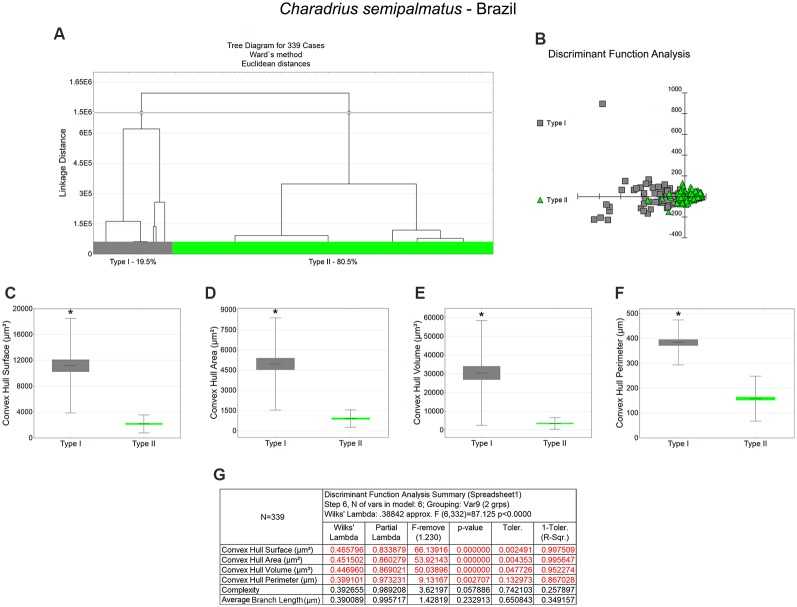
Cluster analysis to classify the morphological phenotypes of radial astrocytes of the hippocampal V area of wintering *C. semipalmatus*. Hierarchical cluster analysis (Ward’s method) was performed after 3D reconstruction of astrocytes from 5 birds. **(A)** Dendrogram groupings of 339 radial astrocytes identified two main morphological phenotypes, Type I and Type II. **(B)** Graphic representation of discriminant analysis. Note higher dispersion of gray-filled squares corresponding to Type I astrocytes. **(C–F)** Graphic representations of mean values and corresponding standard deviations (whiskers) and errors (gray and green areas) of convex hull surface, convex hull area, convex hull volume, and convex hull perimeter respectively; (*) indicates significant difference between Type I and Type II astrocytes. **(G)** Discriminant statistical analysis results. The variables that contributed the most to cluster formation were convex hull surface, convex hull area, and convex hull volume (*p* < 0.000000; see discriminant function analysis summary, **G**). Type I radial astrocytes (gray-filled squares) showed higher x–y dispersion than Type II radial astrocytes (green-filled triangles). Radial astrocytes were reconstructed from the rostral to the caudal regions of the hippocampal formation; cluster analysis was based on multimodal morphometric features of the astrocytes (MMI > 0.55).

Based on multimodal 3D morphological features (MMI > 0.55), we searched for morphological families of radial astrocytes using hierarchical cluster analysis. Independent of the origin of the sample (migrating birds captured in the Bay of Fundy, Canada, or wintering birds captured at Isla Canela in Bragança, Brazil), the results showed two families of astrocytes that we designated as Type I and Type II, respectively, in both *C. pusilla* (Figures 3, 4 and Tables s 1, 2) and *C. semipalmatus* (Figures 5, 6 and Tables s 3, 4).

**Table 1:** Mean values, standard deviation, corresponding standard errors, and significant differences between Type I and Type II radial astrocytes of the hippocampal V area (see also Figure 3).

	*Calidris pusilla*—Canada					
	Complexity		Convex hull surface (μm^2^)		Segments/mm	
	Type I	Type II	Type I	Type II	Type I	Type II
Mean	640.81	257.36	3,481.36	1,210.62	18.75	26.94
SE	75.33	16.60	230.83	31.56	1.76	1.15
SD	573.72	231.28	1,757.98	439.60	13.43	16.02
Test	Mann–Whitney Z (*U*) = 6.4468; *p* < 0.0001		Mann–Whitney Z (*U*) = 11.2634; *p* < 0.0001		Test t—t = 3.5409; *p* = 0.0006	
	**Total branch length (μm)**		**Number of trees**		**Convex hull volume (μm^3^)**	
	**Type I**	**Type II**	**Type I**	**Type II**	**Type I**	**Type II**
Mean	162.73	86.34	1.10	1.08	7,146.89	1,450.35
SE	5.64	1.64	0.04	0.02	812.74	51.47
SD	42.56	22.77	0.31	0.27	6,136.02	715.04
Test	Mann–Whitney Z (*U*) = 10.851; *p* < 0.0001		Test t—t = 0.6297; *p* = 0.5301		Mann–Whitney Z (*U*) = 11.364; *p* < 0.0001	
	**Convex hull area (μm^2^)**		**Convex hull perimeter (μm)**	
	**Type I**	**Type II**	**Type I**	**Type II**
Mean	1,366.12	466.71	229.54	129.00
SE	100.28	13.82	9.67	2.59
SD	757.08	191.97	73.04	35.97
Test	Mann–Whitney Z (*U*) = 10.765; *p* < 0.0001		Mann–Whitney Z (*U*) = 9.7298; *p* < 0.0001	

**Table 2:** Mean values, standard deviation, and corresponding standard errors and significant differences between Type I and Type II radial astrocytes in Figure 4.

	*Calidris pusilla*—Brazil					
	Convex hull surface (μm^2^)		Convex hull area (μm^2^)		Total branch length (μm)	
	Type I	Type II	Type I	Type II	Type I	Type II
Mean	7,076.74	1,664.36	3,018.86	678.18	232.24	99.50
SE	442.39	70.62	230.36	31.98	7.95	2.70
SD	2,255.77	1,040.23	1,174.62	471.08	40.54	39.76
Test	Mann–Whitney Z (*U*) = 8.2614; *p* < 0.0001		Mann–Whitney Z (*U*) = 7.9939; *p* < 0.0001		Test t—t = 16.048; *p* < 0.0001	
	**Convex hull volume (μm^3^)**		**Complexity**		**Average branch length (μm)**	
	**Type I**	**Type II**	**Type I**	**Type II**	**Type I**	**Type II**
Mean	15,061.95	1,949.59	528.88	169.08	188	86.91
SE	1,348.01	108.61	121.11	13.29	14.40	2.93
SD	6,873.50	1,599.86	617.53	195.82	71.99	43
Test	Mann–Whitney Z (*U*) = 8.3267; *p* < 0.0001		Mann–Whitney Z (*U*) = 6.4468; *p* < 0.0001		Mann–Whitney Z (*U*) = 5.8328; *p* < 0.0001	
	**Tortuosity**		**Branch volume (μm^3^)**		**Convex hull perimeter (μm)**	
	**Type I**	**Type II**	**Type I**	**Type II**	**Type I**	**Type II**
Mean	1.35	1.29	37.74	17.15	345.65	166.01
SE	0.04	0.01	3.16	0.73	12.36	4.70
SD	0.19	0.20	15.81	10.70	61.78	69.14
Test			Mann–Whitney Z (*U*) = 6.2717; *p* < 0.0001		Mann–Whitney Z (*U*) = 7.7366; *p* < 0.0001	
	**Segments/mm**		**Surface area (μm^2^)**		**Number of segments**	
	**Type I**	**Type II**	**Type I**	**Type II**	**Type I**	**Type II**
Mean	7.17	15.58	323.75	138.79	1.69	1.34
SE	1.08	0.75	14.75	4.20	0.28	0.06
SD	5.42	11.04	73.76	61.72	1.38	0.89
Test	Mann–Whitney Z (*U*) = 5.8328; *p* < 0.0001		Mann–Whitney Z (*U*) = 7.7721; *p* < 0.0001		Test t—t = 1.2618; *p* = 0.2177	

**Table 3:** Mean values, standard deviation, and corresponding standard errors and significant differences between Type I and Type II radial astrocytes of hippocampal V area (Figure 5).

	*Charadrius semipalmatus—Canada*					
	Complexity		Convex hull surface (μm^2^)		Convex hull volume (μm^3^)	
	Type I	Type II	Type I	Type II	Type I	Type II
Mean	751.73	292.40	2,902.15	903.6	6,134.04	1,025.73
SE	83.82	22.95	506.82	36.07	561.89	68.49
SD	574.63	314.64	1,402.73	493.21	3,852.13	939.09
Test	Mann–Whitney Z (*U*) = 6.3594; *p* < 0.0001		Mann–Whitney Z (*U*) = 10.2504; *p* < 0.0001		Mann–Whitney Z (*U*) = 10.4831; *p* < 0.0001	
	**Convex hull area (μm^2^)**		**Average branch length (μm)**	
	**Type I**	**Type II**	**Type I**	**Type II**
Mean	1,085.40	339.04	63.06	39.31
SE	89.55	14.14	7.37	1.84
SD	613.90	193.91	50.51	25.18
Test	Mann–Whitney Z (*U*) = 9.5847; *p* < 0.0001		Mann–Whitney Z (*U*) = 3.6307; *p* = 0.0003	

**Table 4:** Mean values, standard deviation, corresponding standard errors and significant differences between Type I and Type II radial astrocytes of hippocampal V area (Figure 6).

	*Charadrius semipalmatus*—Brazil					
	Convex hull surface (μm^2^)		Convex hull area (μm^2^)		Convex hull volume (μm^3^)	
	Type I	Type II	Type I	Type II	Type I	Type II
Mean	11,198.97	2,179.08	4,972.80	910.29	30,509.48	3,518.55
SE	900.45	84.47	421.76	38.97	3,446.10	187.31
SD	7,315.28	1,395.66	3,426.43	643.97	27,996.24	3,094.81
Test	Mann–Whitney Z (*U*) = 12.2967; *p* < 0.0001		Mann–Whitney Z (*U*) = 12.147; *p* < 0.0001		Mann–Whitney Z (*U*) = 12.4759; *p* < 0.0001	
	**Convex hull perimeter (μm)**		**Complexity**		**Branch volume (μm^3^)**	
	**Type I**	**Type II**	**Type I**	**Type II**	**Type I**	**Type II**
Mean	384.11	158.35	4,969.17	1,890.68	79.01	43.45
SE	19.65	3.85	627.27	147.04	5.41	1.40
SD	159.61	63.69	5,095.98	2,429.55	43.97	23.09
Test	Mann–Whitney Z (*U*) = 11.317; *p* < 0.0001		Mann–Whitney Z (*U*) = 6.6032; *p* < 0.0001		Mann–Whitney Z (*U*) = 7.6431; *p* < 0.0001	
	**Average branch length (μm)**		**Surface area (μm^2^)**	
	**Type I**	**Type II**	**Type I**	**Type II**
Mean	54.46	27.67	534.31	262.68
SE	7.23	1.47	26.38	6.72
SD	58.77	24.27	214.28	111.04
Test	Mann–Whitney Z (*U*) = 6.7943; *p* < 0.0001		Mann–Whitney Z (*U*) = 10.408; *p* < 0.0001	

Because the proportions of reconstructed cells were rather large (1068 in total; 494 in migrating birds and 574 in wintering birds, approximately 50 radial glia cells per individual), they reflect the quantitative distribution of Type I and Type II astrocytes in the hippocampal V area of *C. pusilla* and *C. semipalmatus*.

### Morphological Differences Between Type I and Type II Astrocytes

Type I radial astrocytes of hippocampal V areas of *C. pusilla* showed higher mean values of morphological complexity and convex hull surface but fewer segments/mm than Type II astrocytes in migrating birds (Figure 3, Table 1). They also showed higher mean values for complexity, total branch length, and convex hull surface, volume, and area than Type II astrocytes in wintering birds (Figure 4, Table 2). Similarly, Type I radial astrocytes of the hippocampal formation of *C. semipalmatus* showed higher mean values for morphological complexity and convex hull surface than Type II astrocytes in migrating birds (Figure 5, Table 3). In wintering birds, they also had higher significant mean values for convex hull surface, volume, area, and perimeter than those of Type II (Figure 6, Table 4).

Because of the random and systematic sampling strategy, we assumed that the selection of radial astrocytes was made without bias and that the total number reconstructed of each type was representative of its distribution in the hippocampus V area. Thus, we estimated the proportion of each type of astrocyte in the sample and verified its variation before and after the completed migration (Figures 3–6). This analysis made it clear that Type II radial astrocytes are less influenced by the migratory process than are Type I cells. In fact, the greatest variation in Type II in *C. pusilla* occurred after the migration was complete and did not reach 20%. Type II astrocytes were more frequent than Type I astrocytes, representing more than 70% of the total of the reconstructed astrocytes in both *C. pusilla* and *C. semipalmatus*.

### Contrasting Migratory Routes Influences on Radial Astrocyte Morphology

**Figure 7:**
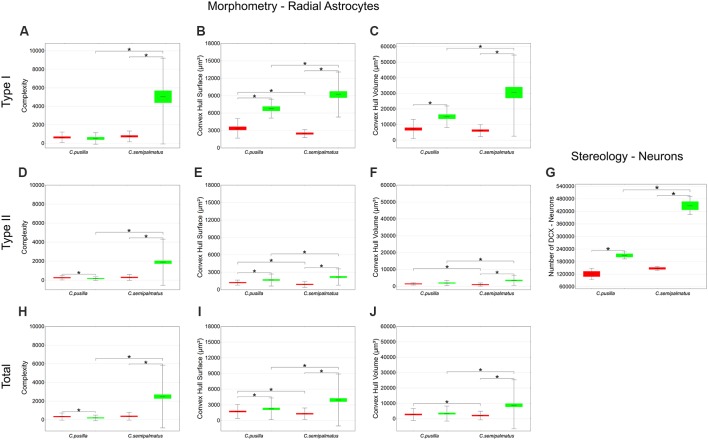
Graphic representations of mean values of morphological complexity **(A,D,H)**, convex hull surface **(B,E,I)**, and convex hull volume **(C,F,J)** and corresponding standard deviations (whiskers) and errors (red and green areas) of hippocampal Type I, Type II, and Total radial astrocytes of hippocampal V area from *C. pusilla* and *C. semipalmatus*, during autumn migration (red) and wintering period (green). The number of DCX-immunolabeled neurons before and after migration **(G)** seems to follow convex hull surface radial astrocyte changes. *Indicates statistical significant differences.

As shown in Figure 7 and Table 5, radial Type I astrocytes from *C. pusilla* and *C. semipalmatus* from individuals captured in the Bay of Fundy, Canada, showed very similar mean values for morphological complexity, convex hull surface, and convex hull volume (A–C). However, we found significant differences in Type I radial astrocytes for convex hull surface and volume between the autumn migratory and wintering birds for both species (Figures 7B,C; Table 5).

**Table 5:** Mean values, standard deviation, corresponding standard errors and significant differences between species comparisons and between Canada and Brazil radial astrocytes of hippocampal V area of *C. pusilla* and *C. semipalmatus* Type I, Type II, Total and number of neurons (Figure 7).

Type I	Complexity				Convex hull surface (μm^2^)				Convex hull volume (μm^3^)			
	*C. pusilla*		*C. semipalmatus*		*C. pusilla*		*C. semipalmatus*		*C. pusilla*		*C. semipalmatus*	
	Canada	Brazil	Canada	Brazil	Canada	Brazil	Canada	Brazil	Canada	Brazil	Canada	Brazil
Mean	640.82	528.88	751.73	4,969.17	3,542.69	6,038.19	2,480.64	9,536.82	7,146.89	15,061.95	6,134.04	30,509.48
SE	75.33	121.11	83.82	627.27	251.61	378.61	105.92	531.22	805.70	1,348.01	561.89	3,446.10
SD	573.72	617.53	574.63	5,095.98	1,882.89	1,815.73	691.54	3,903.66	6,136.02	6,873.50	3,852.13	27,996.24
Test	Kruskal–Wallis *H* = 9.2401; *p* = 0.4923		Kruskal–Wallis *H* = 73.9339; *p* < 0.0001		Kruskal–Wallis *H* = 45.3742; *p* = 0.0003		Kruskal–Wallis *H* = 100.201; *p* < 0.0001		Kruskal–Wallis *H* = 61.4191; *p* < 0.0001		Kruskal–Wallis *H* = 98.3798; *p* < 0.0001	
	**Canada**		**Brazil**		**Canada**		**Brazil**		**Canada**		**Brazil**	
	*C. pusilla*	*C. semipalmatus*	*C. pusilla*	*C. semipalmatus*	*C. pusilla*	*C. semipalmatus*	*C. pusilla*	*C. semipalmatus*	*C. pusilla*	*C. semipalmatus*	*C. pusilla*	*C. semipalmatus*
Test	Kruskal–Wallis *H* = 10.2223; *p* = 0.3609		Kruskal–Wallis *H* = 93.3963; *p* < 0.0001		Kruskal–Wallis *H* = 25.8929; *p* = 0.0123		Kruskal–Wallis *H* = 28.934; *p* = 0.0218		Kruskal–Wallis *H* = 5.2847; *p* = 0.6367		Kruskal–Wallis *H* = 31.676; *p* = 0.0164	
**Type II**	**Complexity**				**Convex hull surface (μm^2^)**				**Convex hull volume (μm^3^)**			
	***C. pusilla***		***C. semipalmatus***		***C. pusilla***		***C. semipalmatus***		***C. pusilla***		***C. semipalmatus***	
	**Canada**	**Brazil**	**Canada**	**Brazil**	**Canada**	**Brazil**	**Canada**	**Brazil**	**Canada**	**Brazil**	**Canada**	**Brazil**
Mean	257.36	169.08	292.40	1,890.68	1,210.62	1,664.36	903.60	2,179.08	1,450.35	1,949.59	1,025.73	3,518.55
SE	16.60	13.32	22.95	147.04	31.56	70.78	35.97	84.47	51.34	108.86	68.49	187.31
SD	231.28	195.82	314.64	2,429.55	439.60	1,040.23	493.21	1,395.66	715.04	1,599.86	939.09	3,094.81
Test	Kruskal–Wallis *H* = 75.6216; *p* = 0.0024		Kruskal–Wallis *H* = 321.744; *p* < 0.0001		Kruskal–Wallis *H* = 85.0756; *p* = 0.0006		Kruskal–Wallis *H* = 326.2974; *p* < 0.0001		Kruskal–Wallis *H* = 38.3014; *p* = 0.1238		Kruskal–Wallis *H* = 331.9798; *p* < 0.0001	
	**Canada**		**Brazil**		**Canada**		**Brazil**		**Canada**		**Brazil**	
	*C. pusilla*	*C. semipalmatus*	*C. pusilla*	*C. semipalmatus*	*C. pusilla*	*C. semipalmatus*	*C. pusilla*	*C. semipalmatus*	*C. pusilla*	*C. semipalmatus*	*C. pusilla*	*C. semipalmatus*
Test	Kruskal–Wallis *H* = 11.7042; *p* = 0.6494		Kruskal–Wallis *H* = 385.662; *p* < 0.0001		Kruskal–Wallis *H* = 129.6746; *p* < 0.0001		Kruskal–Wallis *H* = 111.6071; *p* < 0.0001		Kruskal–Wallis *H* = 128.653; *p* < 0.0001		Kruskal–Wallis *H* = 165.0255; *p* < 0.0001	
**Total**	**Complexity**				**Convex hull surface (μm^2^)**				**Convex hull volume (μm^3^)**			
***C. pusilla***		***C. semipalmatus***		***C. pusilla***		***C. semipalmatus***		***C. pusilla***		***C. semipalmatus***	
**Canada**	**Brazil**	**Canada**	**Brazil**	**Canada**	**Brazil**	**Canada**	**Brazil**	**Canada**	**Brazil**	**Canada**	**Brazil**
Mean	345.61	207.74	384.26	2,490.04	1,512.43	1,995.36	1,303.31	3,935.17	2761.46	3,358.35	2,047.39	8,773.42
SE	23.74	18.88	27.52	182.04	50.93	101.43	72.140	269.67	241.65	313.21	182.36	897.38
SD	376.87	293.65	421.81	3,351.78	789.05	1,551.53	1,105.90	4,965.09	3,836.14	4,872.41	2,795.50	16,522.54
Test	Kruskal–Wallis *H* = 110.6126; *p* < 0.0001		Kruskal–Wallis *H* = 366.2875; *p* < 0.0001		Kruskal–Wallis *H* = 52.3052; *p* = 0.0392		Kruskal–Wallis *H* = 312.1174; *p* < 0.0001		Kruskal–Wallis *H* = 0.2206; *p* = 0.9931		Kruskal–Wallis *H* = 311.143; *p* < 0.0001	
	**Canada**		**Brazil**		**Canada**		**Brazil**		**Canada**		**Brazil**	
	***C. pusilla***	***C. semipalmatus***	***C. pusilla***	***C. semipalmatus***	***C. pusilla***	***C. semipalmatus***	***C. pusilla***	***C. semipalmatus***	***C. pusilla***	***C. semipalmatus***	***C. pusilla***	***C. semipalmatus***
Test	Kruskal–Wallis *H* = 4.9959; *p* = 0.8458		Kruskal–Wallis *H* = 471.9043; *p* < 0.0001		Kruskal–Wallis *H* = 99.6953; *p* < 0.0001		Kruskal–Wallis *H* = 160.1168; *p* < 0.0001		Kruskal–Wallis *H* = 110.1917; *p* < 0.0001		Kruskal–Wallis *H* = 201.1719; *p* < 0.0001	
**Stereology**	**Number of DCX+ (neurons)**			
	***C. pusilla***		***C. semipalmatus***	
	**Canada**	**Brazil**	**Canada**	**Brazil**
Mean	120,874	209,585	147,312	447,567								
SE	12,125	7,441	4,137	19,118								
SD	27,113	16,639	9,251	42,749								
Test	Kruskal–Wallis *H* = 9; *p* = 0.0162		Kruskal–Wallis *H* = 11; *p* = 0.0033	
	**Canada**		**Brazil**									
	***C. pusilla***	***C. semipalmatus***	***C. pusilla***	***C. semipalmatus***								
Test	Kruskal–Wallis *H* = 3; *p* = 0.4227		Kruskal–Wallis *H* = 16; *p* < 0.0001	

Type II radial astrocytes showed different changes in morphological complexity after migration: *C. pusilla* Type II radial astrocytes decreased in complexity, whereas *C. semipalmatus* significantly increased in complexity (Figure 7D). In both species, Type II radial astrocytes increased convex hull surface after migration (Figure 7E), but only *C. semipalmatus* increased in convex hull volume (Figure 7F).

Thus, comparisons of hippocampal Type I and Type II radial astrocytes between individuals captured in the Bay of Fundy, Canada, and those captured on Isle Canela, Bragança, Brazil, showed differential effects of migration and overwintering on radial astrocyte morphology. *C. semipalmatus* tended to show quite marked increases in radial astrocyte morphological complexity, convex hull surface, and volume in wintering birds, whereas such changes were smaller, absent, or even reversed in the direction of change in *C. pusilla*. These species differences may induce distinct effects on radial astrocyte morphology based on the contrasting migratory patterns of the two species. However, the number of DCX-positive new neurons increased after autumnal migration in both species, although to a greater extent in *C. semipalmatus* (Figure 7J).

Between-species comparisons for Type I cells showed significant differences in morphological complexity after migration (Brazil) but not before (Canada). Convex hull surface between-species comparisons showed significant differences before and after migration, and convex hull volume showed significant differences only after migration. Between-species comparisons of Type II cells showed significant differences in morphological complexity only after migration, whereas convex hull surface and volume showed significant differences both before and after migration. Although the species did not differ significantly in the number of DCX-immunolabeled neurons before migration, they did so in the wintering birds (see Figure 7 and Table 5). No significant increase in hippocampal formation volume after migration accompanied these numerical and morphological changes in either species.

**Figure 8:**
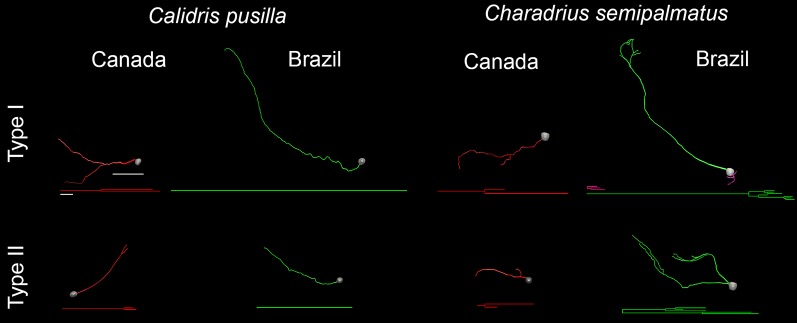
3D reconstructions and corresponding dendrograms of Type I and Type II hippocampal radial astrocytes from the hippocampal V area of *C. pusilla* and *C. semipalmatus* migrating (Canada) and wintering (Brazil) birds. Branches of the same parental (primary branch) trunk are shown in the same color. The 3D drawings were taken from hippocampal radial astrocytes with morphometric features closest to that of the representative “average” cell of each group. 3D cells used to illustrate the average radial astrocyte types were selected from the distance matrix used to obtain the sum of the distances of each cell relative to all others. The cell that best represents a group had the smallest sum of distances. Scale bars: 10 μm for dendrograms and 25 μm for 3D-reconstructed cells.

These changes are also reflected in the 3D reconstructions of representative cells taken from hippocampal radial astrocytes in each group. Figure 8 highlights the main morphological differences between Type I and Type II radial astrocytes in *C. pusilla* and *C. semipalmatus*. Type I radial astrocyte morphological changes after migration seemed to be greater than those of Type II in both species. In contrast, Type II radial astrocytes of *C. pusilla* exhibited less morphological change than those of *C. semipalmatus* after autumnal migration.

### Convex Hull Surface of Radial Astrocytes and Neurogenesis

**Figure 9:**
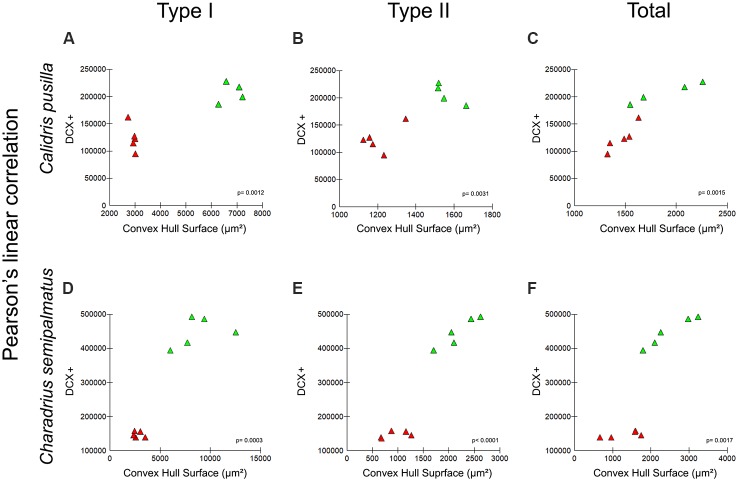
**(A–F)** Graphic representations of Pearson’s linear correlation between the convex hull surface of radial astrocytes of the hippocampal V area and the number of DCX-immunolabeled neurons of the hippocampal formation of both migrating and wintering birds of both *C. pusilla* **(A–C)** and *C. semipalmatus* **(D–F)**. Comparative analysis of Pearson’s linear correlation for Type I, Type II, and total convex hull surface and DCX-immunolabeled neurons before (Canada—red triangles) and after (Brazil—green triangles) migration. Correlation analysis results: Type I, *R*^2^ = 0.89, *p* = 0.001; Type II, *R*^2^ = 0.86, *p* = 0.003; Total, *R*^2^ = 0.88, *p* = 0.001.

Figures 9A–C show graphic representations of Pearson’s linear correlation results between convex hull surface of radial astrocytes of the hippocampal V area and the number of DCX-immunolabeled neurons in the hippocampal formation of the two species. Comparative analysis of results of Pearson’s linear correlation for Type I, Type II, and total convex hull surface and DCX-immunolabeled neurons before (Canada—red dots) and after migration (Brazil—green dots) revealed significant results for both species: Type I, *R^2^* = 0.89, *p* = 0.001; Type II, *R*^2^ = 0.86, *p* = 0.003; total, *R*^2^ = 0.88, *p* = 0.001. However, the main effect on neurogenesis occurred after migration, with an increased number of DCX-immunolabeled neurons after autumnal migration in both species, although to a greater extent in *C. semipalmatus*. In both *C. pusilla* (*R*^2^ = 0.98 *p* = 0.0099) and *C. semipalmatus* (*R*^2^ = 0.9512 *p* = 0.0046), the convex hull surface of total radial astrocytes after migration showed stronger correlations with neurogenesis.

## Discussion

On average, we found that compared with migrating birds, the morphological complexity of RGL α cells in wintering birds significantly increased in *C. semipalmatus* and decreased in *C. pusilla*, although to different magnitudes. These changes were associated with significant increases in the total number of DCX-positive new neurons in both species. We suggest that the non-stop flight of *C. pusilla* and the migratory flight of *C. semipalmatus* with stopovers for feeding and rest differentially affected radial astrocyte morphology and neurogenesis.

### Migration, Neurogenesis, and Radial Astrocyte Morphology

Previous studies have shown that adult brain radial astrocytes can serve as pathways for migration of new neurons toward central nervous system areas where they should potentially enter (Marin and Rubenstein, 2003; Rakic, 2003; Falk and Götz, 2017; Oppenheim, 2019). Based on this evidence, we assumed that changes in radial astrocyte morphology would be equivalent to changes in the orientation, distribution, and number of these tracks in the cerebral parenchyma and would reflect an increase or decrease in neurogenesis. To measure these changes and test the hypothesis that the migratory process could induce them, we used 3D microscopic reconstruction of the branches of these cells and counted the number of DCX-immunolabeled neurons before and after migration. We found a significant correlation between the convex hull surface and the number of DCX-immunolabeled neurons in both *C. pusilla* and *C. semipalmatus* after autumn migration. Our expectation was that differences in winter behaviors between the two species could differentially affect radial astrocyte morphology and neurogenesis. Although our findings suggest as much, we must remember that the glial morphologies and neurogenesis of birds collected in August in the Bay of Fundy and November to March on Isla Canela may differ for other reasons. The birds had spent at least 1 month (September to November) and perhaps as many as 7 months (August to March) in Brazil, so we must consider other possible causes for the significant differences in glial morphology and neurogenesis. That said, the environment through which the birds fly changes dramatically and their journeys may be visuospatially enriched and engage perceptual processes involved in celestial, olfactory, and geomagnetic navigation in ways that do not occur outside the period of migration (Biro et al., 2004; Bingman and Cheng, 2005; Frost and Mouritsen, 2006; Thorup and Holland, 2009; Mouritsen et al., 2016). Thus, we suggest that migration may be considered as a kind of environmental enrichment that could contribute to increased radial astrocyte convex hull surface and neurogenesis.

We demonstrated significant differential effects on *C. pusilla* and *C. semipalmatus* hippocampal V radial astrocyte morphology and neurogenesis after autumn migration, but why hippocampal neurogenesis and radial astrocyte morphology might differ between wintering and actively migrating birds is unclear. Cognitive activity, environmental enrichment, diet, and stress all affect levels of hippocampal neurogenesis. Indeed, as indicated previously (de Morais Magalhães et al., 2017), a diet high in polyunsaturated fatty acids (PUFAs) leads to less hippocampal neurogenesis than does a diet low in PUFAs (Hall et al., 2014). During their stopover in the Bay of Fundy, semipalmated sandpipers and semipalmated plovers are exposed to a diet that is extremely high in PUFAs (Maillet and Weber, 2007; Nagahuedi et al., 2009; Weber, 2009). As previously shown (Hall et al., 2014) and as confirmed for *C. pusilla* (de Morais Magalhães et al., 2017), diet during this stopover includes large amounts of the amphipod *Corophium volutator* in which 45% of total lipids are PUFAs, which may, therefore, decrease hippocampal neurogenesis during stopover.

Similarly, stress and elevated glucocorticoid levels reduce hippocampal neurogenesis (Barnea and Pravosudov, 2011; Aimone et al., 2014; Cameron and Glover, 2015; LaDage, 2015). The glucocorticoid hormone corticosterone is elevated in long-distance migrants in preparation for migration, including accumulating fat reserves for migration and during refueling stopovers (Piersma et al., 2000; O’Reilly and Wingfield, 2003; Eikenaar et al., 2014). Increased spatial processing is also associated with an increase in the number of new neurons in the hippocampus (Gould et al., 1999; Ambrogini et al., 2000; Döbrössy et al., 2003; Hairston et al., 2005; LaDage et al., 2010), and bird behavior during migration is consistent with elevated demands on spatial learning and memory (LaDage et al., 2011). In addition, studies in rats and mice have shown that environmental enrichment is associated with elevated neurogenesis and neuronal recruitment in the dentate gyrus (Kempermann et al., 2010; Bednarczyk et al., 2011; Mustroph et al., 2012; Bechara and Kelly, 2013; Birch et al., 2013; Grégoire et al., 2014).

It is clear from Figure 9 that the number of DCX-positive newly generated neurons after migration was greater in *C. semipalmatus* than in *C. pusilla*. We suggest that strenuous exercise inducing elevated glucocorticoid levels and the less visually enriched environment during uninterrupted transatlantic flight may be responsible for these lower levels of hippocampal neurogenesis found in semipalmated sandpipers collected in Isle Canela. In contrast, *C. semipalmatus* individuals flew overland with multiple stopovers for rest and feeding, under the influence of frequent environmental changes along the journey and were not subjected to the strenuous exercise associated with transatlantic flight. They, therefore, might be expected to show greater neurogenesis than *C. pusilla* after migration.

Because the long flight differentially affected Type I compared to Type II astrocytes in both *C. pusilla* and *C. semipalmatus*, we suggest that these cells may have distinct physiological roles. At least in *C. pusilla*, previous findings (Carvalho-Paulo et al., 2017) suggest a greater percentage of Type II astrocytes (72.5%) interacting with blood vessels compared to Type I astrocytes (27.5%) in both migrating and wintering birds. Because this interaction may reflect their relative contribution to the neurovascular unit, these authors hypothesized that Type II astrocytes may be more involved in the neurovascular unit than are Type I astrocytes.

### Similarities and Differences in Migrating and Wintering Environmental Influences: Methodological Limitations

Although we have emphasized the contrasting migratory flights as the most relevant factor for the morphological changes observed in the radial glia, we also note that the capture of the birds in the Bay of Fundy occurred in August and that the birds in the wintering period were captured on Canela Island between August and May. Thus, for some captured birds, the transatlantic flight may have occurred at least 9 months earlier, and during that time, several other factors may have contributed to the observed morphological changes. We list seven factors that may down- or upregulate neurogenesis and change radial astrocyte morphology: (1) long-distance uninterrupted transatlantic flight vs. interrupted overland flight with resting and feeding; (2) time windows of capture (collection in August in the Bay of Fundy and from August to May on Isla Canela); (3) environmental differences along the journey; (4) differences in available nutrients along the journey and on the Bay of Fundy and Isle Canela; (5) post-breeding physiological condition in the Bay of Fundy vs. wintering, or even pre-breeding physiological condition in Brazil; (6) very long days in the Arctic summer vs. 12-h days at the equator; and (7) contrasting visual stimuli between the Bay of Fundy and Isle Canela environments.

Because the two species had similar diets in the Bay of Fundy and in wintering areas, shared similar pre- and post-breeding physiological conditions in the Bay of Fundy and Brazil, were exposed to similar day lengths both in the Arctic summer and at the equator, and shared similar visual stimuli in both Bay of Fundy (Canada) and Isle Canela (Brazil), it is not likely that these parameters could cause contrasting morphological changes in radial astrocyte arbors and neurogenesis. Thus, the evidence suggests that, phylogenetic distinctions apart, it is reasonable to associate these differences with overland interrupted flight for resting and feeding compared to uninterrupted transatlantic 5-day flights.

Cognitive activity, environmental enrichment, diet, and metabolic changes all affect astrocyte morphophysiology. *C. pusilla* and* C. semipalmatus* were exposed to these differentially because of their contrasting migratory flights, so it is reasonable that these factors may have significantly contributed to the differential effects. In line with this view, previous studies in rats and mice demonstrate a significant influence of environmental changes on morphological plasticity and number of hippocampal astrocytes (Soffié et al., 1999; Viola et al., 2009; Diniz et al., 2010, 2012, 2016; Rodríguez et al., 2014; Sampedro-Piquero et al., 2014; Yeh et al., 2015; Salois and Smith, 2016; Tsai et al., 2016; Verkhratsky et al., 2016). Additionally, as compared with the *C. semipalmatus* overland migratory pathway, there is another possible interpretation of less intense use of the hippocampus during the transatlantic journey of *C. pusilla*. If compared to overland continental migration, fall migration over the Atlantic Ocean relies more on a compass sense and less on processing of spatial features, less hippocampal involvement and perhaps less hippocampal neurogenesis might be expected in *C. pusilla* compared to *C. semipalmatus*. Less visual-spatial stimuli and landmark recognition and reduced spatial memory demands would be predicted on the transatlantic route compared to an overland continental route with multiple stopovers.

Thus, as we have previously discussed (de Morais Magalhães et al., 2017), if long-distance migration does act to upregulate neurogenesis, its effects on *C. pusilla* would be seen not during migration but during the wintering period that follows. In contrast, in *C. semipalmatus*, where stress levels seem to be less intense and environmental enrichment higher than that of *C. pusilla*, long-distance migration may promptly upregulate neurogenesis.

We have noted the lack of published findings on the potential influence of sex and age on hippocampal radial astrocyte morphology in long-distance migratory birds (Carvalho-Paulo et al., 2017). Because of this lack and because we did not measure the age of individuals in our sample for technical reasons, it is difficult to discuss these potential influences in detail. Previous reports have demonstrated the influence of experience and sex on hippocampus-dependent tasks in birds (Astié et al., 2015; Rensel et al., 2015; Guigueno et al., 2016; Bingman and MacDougall-Shackleton, 2017). In addition, migratory behavior is accompanied by hippocampal morphological changes including volume and neurogenesis (Barkan et al., 2016, 2017; de Morais Magalhães et al., 2017).

Because environmental inputs are associated with migration and many other factors influence neurogenesis, comparing more groups of birds at different stages of their wintering period is an important next step. Documenting neurogenesis and morphological and numerical changes in radial glia between departure from the northern hemisphere and the wintering period in the southern hemisphere may shed light on radial astrocyte and neuron regulation in many species. Our study was limited to a group caught in Canada at the beginning of migration and a second group caught in Brazil during wintering. However, the clear differences we observed between migrating and wintering birds indicate that long-distance migratory shorebirds may offer an opportunity for addressing many questions about the natural control and function of adult hippocampal neurogenesis.

## Data Availability

The raw data supporting the conclusions of this manuscript will be made available by the authors, without undue reservation, to any qualified researcher.

## Ethics Statement

The animal study was reviewed and approved by the Animal Users Subcommittee of the University of Western Ontario and the Federal University of Pará.

## Author Contributions

All listed authors contributed substantially to the conception or design of the work; the acquisition, analysis, or interpretation of data for the work; drafting the work or revising it critically for important intellectual content; and/or final approval of the version to be published; and agreed to be accountable for all aspects of the work in ensuring that questions related to the accuracy or integrity of any part of the work are appropriately investigated and resolved.

## Conflict of Interest Statement

The authors declare that the research was conducted in the absence of any commercial or financial relationships that could be construed as a potential conflict of interest.
