# Supplementary material for: Differential Change in Hippocampal Radial Astrocytes and Neurogenesis in Shorebirds With Contrasting Migratory Routes
Source: Front Neuroanat. 2019 Sep 25;13:82. doi: 10.3389/fnana.2019.00082 (PMC6798042; doi:10.3389/fnana.2019.00082)
Supplement: Supplementary file 1 [file Data_Sheet_1.docx]

Table S1. Stereological parameters for DCX-immunopositive neurons in the right and left hippocampal formations of *Calidris pusilla*.

| **Canada** | **a (frame)**  **(µm)** | **A (x, y step)**  **(µm)** | **N° sections** | **N° counting**  **frames** | **ASF** | **SSF** | **TSF** | **ΣQ‐** |
| --- | --- | --- | --- | --- | --- | --- | --- | --- |
| Right hippocampal formation | | | | | | | | |
| *C. pusilla* A | 65 × 65 | 300 × 300 | 10 | 118 | 0.0469 | 0.166 | 0.60 | 291 |
| *C. pusilla* B | 65 × 65 | 300 × 300 | 10 | 115 | 0.0469 | 0.166 | 0.60 | 286 |
| *C. pusilla* C | 65 × 65 | 300 × 300 | 10 | 85 | 0.0469 | 0.166 | 0.63 | 238 |
| *C. pusilla* D | 65 × 65 | 300 × 300 | 10 | 141 | 0.0469 | 0.166 | 0.63 | 274 |
| *C. pusilla* E | 65 × 65 | 300 × 300 | 10 | 103 | 0.0469 | 0.166 | 0.68 | 300 |
| Left hippocampal formation | | | | | | | | |
| *C. pusilla* A | 65 × 65 | 300 × 300 | 10 | 111 | 0.0469 | 0.166 | 0.56 | 306 |
| *C. pusilla* B | 65 × 65 | 300 × 300 | 10 | 114 | 0.0469 | 0.166 | 0.60 | 281 |
| *C. pusilla* C | 65 × 65 | 300 × 300 | 10 | 79 | 0.0469 | 0.166 | 0.62 | 213 |
| *C. pusilla* D | 65 × 65 | 300 × 300 | 10 | 153 | 0.0469 | 0.166 | 0.66 | 297 |
| *C. pusilla* E | 65 × 65 | 300 × 300 | 10 | 123 | 0.0469 | 0.166 | 0.69 | 368 |

| **Brazil** | **a (frame)**  **(µm)** | **A (x, y step)**  **(µm)** | **N° sections** | **N° counting**  **frames** | **ASF** | **SSF** | **TSF** | **ΣQ‐** |
| --- | --- | --- | --- | --- | --- | --- | --- | --- |
| Right hippocampal formation | | | | | | | | |
| *C. pusilla* F | 65 × 65 | 250 × 250 | 10 | 130 | 0.0676 | 0.166 | 0.45 | 449 |
| *C. pusilla* G | 65 × 65 | 300 × 300 | 10 | 117 | 0.0469 | 0.166 | 0.47 | 348 |
| *C. pusilla* H | 65 × 65 | 250 × 250 | 10 | 186 | 0.0676 | 0.166 | 0.51 | 560 |
| *C. pusilla* I | 65 × 65 | 300 × 300 | 10 | 146 | 0.0469 | 0.166 | 0.48 | 479 |
| *C. pusilla* J | 65 × 65 | 300 × 300 | 10 | 143 | 0.0469 | 0.166 | 0.58 | 377 |
| Left hippocampal formation | | | | | | | | |
| *C. pusilla* F | 65 × 65 | 250 × 250 | 10 | 149 | 0.0676 | 0.166 | 0.45 | 578 |
| *C. pusilla* G | 65 × 65 | 300 × 300 | 10 | 131 | 0.0469 | 0.166 | 0.49 | 440 |
| *C. pusilla* H | 65 × 65 | 250 × 250 | 10 | 170 | 0.0676 | 0.166 | 0.50 | 529 |
| *C. pusilla* I | 65 × 65 | 300 × 300 | 10 | 113 | 0.0469 | 0.166 | 0.48 | 374 |
| *C. pusilla* J | 65 × 65 | 300 × 300 | 10 | 140 | 0.0469 | 0.166 | 0.58 | 461 |

ΣQ- = total number of objects of interest counted using the optical dissector; SSF = section sampling fraction; ASF = area sampling fraction; TSF = thickness sampling fraction; a (frame) = area of counting frame; A (x, y step) = grid size. “Canada” refers to birds collected during autumn migration in the Bay of Fundy, and “Brazil” refers to wintering birds collected at Isla Canela.

Table S2. Stereological parameters for DCX-immunopositive neurons in the right and left hippocampal formations of *Charadrius semipalmatus*.

| **Canada** | **a (frame)**  **(µm)** | **A (x, y step)**  **(µm)** | **N° sections** | **N° counting**  **frames** | **ASF** | **SSF** | **TSF** | **ΣQ‐** |
| --- | --- | --- | --- | --- | --- | --- | --- | --- |
| Right hippocampal formation | | | | | | | | |
| *C. semipalmatus* A | 65 × 65 | 300 × 300 | 10 | 156 | 0.0469 | 0.166 | 0.65 | 366 |
| *C. semipalmatus* B | 65 × 65 | 300 × 300 | 10 | 200 | 0.0469 | 0.166 | 0.64 | 354 |
| *C. semipalmatus* C | 65 × 65 | 300 × 300 | 10 | 197 | 0.0469 | 0.166 | 0.67 | 362 |
| *C. semipalmatus* D | 65 × 65 | 300 × 300 | 10 | 215 | 0.0469 | 0.166 | 0.62 | 414 |
| *C. semipalmatus* E | 65 × 65 | 300 × 300 | 10 | 175 | 0.0469 | 0.166 | 0.66 | 394 |
| Left hippocampal formation | | | | | | | | |
| *C. semipalmatus* A | 65 × 65 | 300 × 300 | 10 | 163 | 0.0469 | 0.166 | 0.65 | 322 |
| *C. semipalmatus* B | 65 × 65 | 300 × 300 | 10 | 207 | 0.0469 | 0.166 | 0.64 | 319 |
| *C. semipalmatus* C | 65 × 65 | 300 × 300 | 10 | 215 | 0.0469 | 0.166 | 0.67 | 374 |
| *C. semipalmatus* D | 65 × 65 | 300 × 300 | 10 | 195 | 0.0469 | 0.166 | 0.63 | 339 |
| *C. semipalmatus* E | 65 × 65 | 300 × 300 | 10 | 202 | 0.0469 | 0.166 | 0.67 | 394 |

| **Brazil** | **a (frame)**  **(µm)** | **A (x, y step)**  **(µm)** | **N° sections** | **N° counting**  **frames** | **ASF** | **SSF** | **TSF** | **ΣQ‐** |
| --- | --- | --- | --- | --- | --- | --- | --- | --- |
| Right hippocampal formation | | | | | | | | |
| *C. semipalmatus* F | 75 × 75 | 200 × 200 | 10 | 633 | 0.1406 | 0.166 | 0.58 | 2721 |
| *C. semipalmatus* G | 75 × 75 | 300 × 300 | 10 | 270 | 0.0625 | 0.166 | 0.64 | 1738 |
| *C. semipalmatus* H | 75 × 75 | 300 × 300 | 10 | 308 | 0.0625 | 0.166 | 0.70 | 1587 |
| *C. semipalmatus* I | 75 × 75 | 300 × 300 | 10 | 275 | 0.0625 | 0.166 | 0.63 | 1496 |
| *C. semipalmatus* J | 65 × 65 | 425 × 425 | 10 | 105 | 0.0233 | 0.166 | 0.62 | 534 |
| Left hippocampal formation | | | | | | | | |
| *C. semipalmatus* F | 75 × 75 | 200 × 200 | 10 | 605 | 0.1406 | 0.166 | 0.58 | 2583 |
| *C. semipalmatus* G | 75 × 75 | 300 × 300 | 10 | 255 | 0.0625 | 0.166 | 0.71 | 1530 |
| *C. semipalmatus* H | 75 × 75 | 300 × 300 | 10 | 300 | 0.0625 | 0.166 | 0.69 | 1577 |
| *C. semipalmatus* I | 75 × 75 | 300 × 300 | 10 | 258 | 0.0625 | 0.166 | 0.64 | 1637 |
| *C. semipalmatus* J | 65 × 65 | 425 × 425 | 10 | 95 | 0.0233 | 0.166 | 0.63 | 476 |

ΣQ- = total number of objects of interest counted using the optical dissector; SSF = section sampling fraction; ASF = area sampling fraction; TSF = thickness sampling fraction; a (frame) = area of counting frame; A (x, y step) = grid size. “Canada” refers to birds collected during autumn migration in the Bay of Fundy, and “Brazil” refers to wintering birds collected at Isla Canela.

Table S3. Stereological results for DCX-immunopositive neurons in the right and left hippocampal formations of Calidris pusilla.

| **Canada** | **Capture date** | **N° of DCX+** | **SCE** | **Thickness**  **(µm)** | **DCX+/mm³** | **N° of DCX+** | **SCE** | **Thickness (µm)** | **DCX+/mm^3^** |
| --- | --- | --- | --- | --- | --- | --- | --- | --- | --- |
|  |  | Right hippocampal formation | | | | Left hippocampal formation | | | |
| *C. pusilla* A | 04/Aug/2012 | 68054 | 0.051 | 26.60 | 12816 | 71037 | 0.051 | 26.80 | 13714 |
| *C. pusilla* B | 04/Aug/2012 | 62038 | 0.050 | 25.27 | 17427 | 60708 | 0.050 | 25.10 | 16958 |
| *C. pusilla* C | 12/Aug/2012 | 49679 | 0.049 | 24.24 | 13143 | 45219 | 0.049 | 24.61 | 11685 |
| *C. pusilla* D | 07/Aug/2012 | 56397 | 0.047 | 24.06 | 10951 | 58654 | 0.047 | 23.14 | 12723 |
| *C. pusilla* E | 07/Aug/2012 | 58099 | 0.048 | 22.16 | 11392 | 69190 | 0.050 | 21.97 | 13567 |
| **Mean** |  | **58853** | **0.049** | **24.47** | **13146** | **60962** | **0.049** | **24.32** | **13729** |
| **S.D.** |  | **6811** | **0.002** | **1.64** | **2565** | **10277** | **0.002** | **1.85** | **1977** |
| CV^2^ |  | 0.01339 |  |  |  | 0.02842 |  |  |  |
| CE^2^ |  | 0.00240 |  |  |  | 0.00244 |  |  |  |
| CE^2^/CV^2^ |  | 0.17928 |  |  |  | 0.08587 |  |  |  |
| CV^2^-CE^2^ |  | 0.01099 |  |  |  | 0.02598 |  |  |  |
| CVB^2^(%CV^2^) |  | 82.07 |  |  |  | 91.41 |  |  |  |
| **Brazil** | **Capture date** | **N° of DCX+** | **SCE** | **Thickness**  **(µm)** | **DCX+/mm³** | **N° of DCX+** | **SCE** | **Thickness (µm)** | **DCX+/mm^3^** |
|  |  | Right hippocampal formation | | | | Left hippocampal formation | | | |
| *C. pusilla* F | 14/Jan/2014 | 97723 | 0.047 | 34.52 | 15293 | 126596 | 0.042 | 34.39 | 19008 |
| *C. pusilla* G | 10/Nov/2014 | 99105 | 0.054 | 32.48 | 18770 | 118437 | 0.048 | 31.02 | 18362 |
| *C. pusilla* H | 10/Nov/2014 | 101195 | 0.042 | 30.24 | 18844 | 98213 | 0.043 | 31.04 | 15739 |
| *C. pusilla* I | 10/Nov/2014 | 130530 | 0.046 | 31.44 | 24863 | 104407 | 0.052 | 31.95 | 16652 |
| *C. pusilla* J | 12/Sep/2015 | 81415 | 0.052 | 25.65 | 21038 | 104544 | 0.047 | 26.57 | 20221 |
| **Mean** |  | **101994** | **0.048** | **30.87** | **19762** | **110440** | **0.046** | **30.99** | **17997** |
| **S.D.** |  | **17783** | **0.005** | **3.31** | **3516** | **11680** | **0.004** | **2.83** | **1803** |
| CV^2^ |  | 0.03040 |  |  |  | 0.01118 |  |  |  |
| CE^2^ |  | 0.00232 |  |  |  | 0.00215 |  |  |  |
| CVB^2^ |  | 0.07642 |  |  |  | 0.19250 |  |  |  |
| CVB^2^/CV^2^ |  | 0.02808 |  |  |  | 0.00903 |  |  |  |
| CVB^2^(%CV^2^) |  | 92.36 |  |  |  | 80.75 |  |  |  |

SCE = Sheaffer coefficient of error; S.D. = standard deviation. “Canada” refers to birds collected during autumn migration in the Bay of Fundy, and “Brazil” refers to wintering birds collected at Isla Canela.

Table S4. Stereological results for DCX-immunopositive neurons in the right and left hippocampal formations of Charadrius semipalmatus.

| **Canada** | **Capture date** | **N° of DCX+** | **SCE** | **Thickness**  **(µm)** | **DCX+/mm³** | **N° of**  **DCX+** | **SCE** | **Thickness (µm)** | **DCX+/mm^3^** |
| --- | --- | --- | --- | --- | --- | --- | --- | --- | --- |
|  |  | Right hippocampal formation | | | | Left hippocampal formation | | | |
| *C. semipalmatus* A | 03/Aug/2012 | 73836 | 0.044 | 23.43 | 6652 | 64291 | 0.043 | 23.24 | 6042 |
| *C. semipalmatus* B | 03/Aug/2012 | 72682 | 0.049 | 23.63 | 5701 | 66457 | 0.044 | 23.86 | 4993 |
| *C. semipalmatus* C | 03/Aug/2012 | 70524 | 0.050 | 22.45 | 7038 | 74381 | 0.043 | 22.60 | 7813 |
| *C. semipalmatus* D | 04/Aug/2012 | 87466 | 0.045 | 24.38 | 6199 | 70720 | 0.045 | 24.17 | 5147 |
| *C. semipalmatus* E | 07/Aug/2012 | 77914 | 0.041 | 22.94 | 5771 | 77952 | 0.040 | 22.70 | 5941 |
| **Mean** |  | **76484** | **0.046** | **23.37** | **6272** | **70760** | **0.043** | **23.31** | **5987** |
| **S.D.** |  | **6701** | **0.004** | **0.73** | **573.19** | **5594.75** | **0.002** | **0.69** | **1121.78** |
| CV |  | 0.00768 |  |  |  | 0.00625 |  |  |  |
| CV^2^ |  | 0.00210 |  |  |  | 0.00185 |  |  |  |
| CE^2^ |  | 0.27323 |  |  |  | 0.29577 |  |  |  |
| CE^2^/CV^2^ |  | 0.00558 |  |  |  | 0.00440 |  |  |  |
| CV^2^-CE^2^ |  | 72.68 |  |  |  | 70.42 |  |  |  |
| **Brazil** | **Capture Date** | **N° of DCX+** | **SCE** | **Thickness**  **(µm)** | **DCX+/mm³** | **N° of**  **DCX +** | **SCE** | **Thickness (µm)** | **DCX+/mm^3^** |
|  |  | Right Hippocampal Formation | | | | Left Hippocampal Formation | | | |
| *C. semipalmatus* F | 20/May/2015 | 202865 | 0.023 | 30.33 | 16793 | 191612 | 0.025 | 30.24 | 16252 |
| *C. semipalmatus* G | 17/Jun/2015 | 266793 | 0.028 | 28.40 | 24818 | 226694 | 0.035 | 26.24 | 21903 |
| *C. semipalmatus* H | 15/Aug/2015 | 224257 | 0.032 | 26.84 | 18534 | 223179 | 0.031 | 29.45 | 19206 |
| *C. semipalmatus* I | 15/Aug/2015 | 234154 | 0.034 | 30.58 | 18823 | 252365 | 0.029 | 32.14 | 20635 |
| *C. semipalmatus* J | 13/Oct/2015 | 223487 | 0.043 | 31.45 | 17667 | 193283 | 0.046 | 30.45 | 12843 |
| **Mean** |  | **230311** | **0.032** | **29.52** | **19327** | **217427** | **0.033** | **29.70** | **18168** |
| **S.D.** |  | **23357** | **0.007** | **1.87** | **3171** | **25441** | **0.008** | **2.17** | **3645** |
| CV^2^ |  | 0.01028 |  |  |  | 0.01369 |  |  |  |
| CE^2^ |  | 0.00102 |  |  |  | 0.00110 |  |  |  |
| CVB^2^ |  | 0.09956 |  |  |  | 0.08051 |  |  |  |
| CVB^2^/CV^2^ |  | 0.00926 |  |  |  | 0.01259 |  |  |  |
| CVB^2^(%CV^2^) |  | 90.04 |  |  |  | 91.95 |  |  |  |

SCE = Sheaffer coefficient of error; S.D. = standard deviation. “Canada” refers to birds collected during autumn migration in the Bay of Fundy, and “Brazil” refers to wintering birds collected at Isla Canela.

Table S5. Volume estimates for the right and left hippocampal formation and telencephalon, and the ratio between them for Calidris pusilla in Canada and Brazil.

| **Canada** | **Estimated vol. (mm³)** | **CE Gundersen m = 1** | **Estimated vol. (mm³)** | **CE Gundersen m = 1** | **Vol.** | **Estimated vol. (mm³)** | **CE Gundersen m = 1** | **Estimated vol. (mm³)** | **CE Gundersen m = 1** | **Vol.** |
| --- | --- | --- | --- | --- | --- | --- | --- | --- | --- | --- |
|  | **RHF** | | **RT** | | **RHF/RT** | **LHF** | | **LT** | | **LHF/LT** |
| *C. pusilla* A | 5.31 | 0.022 | 89.10 | 0.006 | 0.060 | 5.18 | 0.022 | 84.6 | 0.008 | 0.061 |
| *C. pusilla* B | 3.56 | 0.030 | 77.30 | 0.008 | 0.046 | 3.58 | 0.030 | 80.4 | 0.009 | 0.045 |
| *C. pusilla* C | 3.78 | 0.027 | 71.40 | 0.008 | 0.053 | 3.87 | 0.026 | 82.7 | 0.007 | 0.047 |
| *C. pusilla* D | 5.15 | 0.021 | 79.60 | 0.006 | 0.065 | 4.61 | 0.022 | 88.9 | 0.007 | 0.052 |
| *C. pusilla* E | 5.10 | 0.019 | 97.90 | 0.004 | 0.052 | 5.10 | 0.020 | 105.0 | 0.005 | 0.049 |
| **Mean** | **4.58** | **0.024** | **83.06** | **0.006** | **0.055** | **4.47** | **0.024** | **88.32** | **0.007** | **0.051** |
| **S.D.** | **0.84** | **0.004** | **10.46** | **0.002** | **0.007** | **0.72** | **0.004** | **9.83** | **0.001** | **0.007** |
| **Brazil** | **Estimated vol. (mm³)** | **CE Gundersen m = 1** | **Estimated vol. (mm³)** | **CE Gundersen m = 1** | **Vol.** | **Estimated vol. (mm³)** | **CE Gundersen m = 1** | **Estimated vol. (mm³)** | **CE Gundersen m = 1** | **vol.** |
|  | **RHF** | | **RT** | | **RHF/RT** | **LHF** | | **LT** | | **LHF/LT** |
| *C. pusilla* F | 6.39 | 0.019 | 111.00 | 0.007 | 0.058 | 6.66 | 0.022 | 108.00 | 0.008 | 0.061 |
| *C. pusilla* G | 5.28 | 0.025 | 99.20 | 0.006 | 0.053 | 6.45 | 0.024 | 95.30 | 0.005 | 0.067 |
| *C. pusilla* H | 5.37 | 0.029 | 120.00 | 0.006 | 0.045 | 6.24 | 0.026 | 117.00 | 0.006 | 0.053 |
| *C. pusilla* I | 5.25 | 0.025 | 83.10 | 0.011 | 0.063 | 6.27 | 0.023 | 81.90 | 0.011 | 0.076 |
| *C. pusilla* J | 3.87 | 0,016 | 88.98 | 0,006 | 0.043 | 5,17 | 0,013 | 97,42 | 0,005 | 0,053 |
| **Mean** | **5.23** | **0.025** | **100.46** | **0.008** | **0.052** | **6.41** | **0.024** | **100.55** | **0.008** | **0.064** |
| **S.D.** | **0.90** | **0.004** | **15.23** | **0.002** | **0.008** | **0.19** | **0.002** | **15.29** | **0.003** | **0.010** |

CE = coefficient of error; Vol. = volume; RHF = right hippocampal formation; LHF = left hippocampal formation; RT = right telencephalon; LT = left telencephalon; CE = coefficient of error; Vol. = volume. “Canada” refers to birds collected during autumn migration in the Bay of Fundy, and “Brazil” refers to wintering birds collected at Isla Canela.

Table S6. Volume estimates for the right and left hippocampal formation and telencephalon, and the ratio between them for Canada and Brazil Charadrius semipalmatus.

| **Canada** | **Estimated vol. (mm³)** | **CE Gundersen m = 1** | **Estimated vol. (mm³)** | **CE Gundersen m = 1** | **Vol.** | **Estimated vol. (mm³)** | **CE Gundersen m = 1** | **Estimated vol. (mm³)** | **CE Gundersen m = 1** | **Vol.** |
| --- | --- | --- | --- | --- | --- | --- | --- | --- | --- | --- |
|  | **RHF** | | **RT** | | **RHF/RT** | **LHF** | | **LT** | | **LHF/LT** |
| *C. semipalmatus* A | 11.10 | 0.011 | 155.65 | 0.005 | 0.071 | 10.12 | 0.013 | 126.92 | 0.005 | 0.080 |
| *C. semipalmatus* B | 12.75 | 0.009 | 167.67 | 0.003 | 0.076 | 12.25 | 0.010 | 178.86 | 0.008 | 0.068 |
| *C. semipalmatus* C | 10.02 | 0.012 | 135.55 | 0.003 | 0.074 | 10.15 | 0.012 | 134.48 | 0.003 | 0.075 |
| *C. semipalmatus* D | 14.11 | 0.012 | 166.18 | 0.005 | 0.085 | 14.58 | 0.009 | 171.74 | 0.007 | 0.085 |
| *C. semipalmatus* E | 13.50 | 0.009 | 160.78 | 0.004 | 0.084 | 13.50 | 0.010 | 158.98 | 0.002 | 0.085 |
| **Mean** | **12.30** | **0.011** | **157.17** | **0.004** | **0.078** | **12.12** | **0.011** | **154.20** | **0.005** | **0.079** |
| **S.D.** | **1.70** | **0.002** | **12.99** | **0.001** | **0.006** | **1.99** | **0.002** | **22.76** | **0.003** | **0.007** |
| **Brazil** | **Estimated vol. (mm³)** | **CE Gundersen m = 1** | **Estimated vol. (mm³)** | **CE Gundersen m = 1** | **Vol.** | **Estimated vol. (mm³)** | **CE Gundersen m = 1** | **Estimated vol. (mm³)** | **CE Gundersen m = 1** | **Vol.** |
|  | **RHF** | | **RT** | | **RHF/RT** | **LHF** | | **LT** | | **LHF/LT** |
| *C. semipalmatus* F | 12.08 | 0.015 | 175.99 | 0.009 | 0.069 | 11.79 | 0.014 | 154.77 | 0.007 | 0.076 |
| *C. semipalmatus* G | 10.75 | 0.013 | 167.99 | 0.010 | 0.064 | 10.35 | 0.011 | 165.31 | 0.010 | 0.063 |
| *C. semipalmatus* H | 12.10 | 0.014 | 163.70 | 0.006 | 0.074 | 11.62 | 0.013 | 175.10 | 0.008 | 0.066 |
| *C. semipalmatus* I | 12.44 | 0.012 | 165.62 | 0.007 | 0.075 | 12.23 | 0.012 | 155.21 | 0.008 | 0.079 |
| *C. semipalmatus* J | 12.65 | 0.014 | 188.01 | 0.009 | 0.067 | 15.05 | 0.012 | 194.36 | 0.005 | 0.077 |
| **Mean** | **12.00** | **0.014** | **172.26** | **0.008** | **0.070** | **12.21** | **0.012** | **168.95** | **0.008** | **0.072** |
| **S.D.** | **0.74** | **0.001** | **9.97** | **0.002** | **0.005** | **1.74** | **0.001** | **16.48** | **0.002** | **0.007** |

CE = coefficient of error; Vol. = volume; RHF = right hippocampal formation; LHF = left hippocampal formation; RT = right telencephalon; LT = left telencephalon; CE = coefficient of error; Vol. = volume. “Canada” refers to birds collected during autumn migration in the Bay of Fundy, and “Brazil” refers to wintering birds collected at Isla Canela.
